# Tandospirone, a 5-HT1A partial agonist is effective in treating anorexia nervosa: a case series

**DOI:** 10.1186/1744-859X-12-7

**Published:** 2013-03-09

**Authors:** Kyoji Okita, Akihiro Shiina, Michiko Nakazato, Masaomi Iyo

**Affiliations:** 1 Department of Psychiatry, Graduate School of Medicine, Chiba University, 1-8-1 Inohana, Chuo-ku, Chiba, Chiba, 260-8677, Japan; 2 Center for Forensic Mental Health, Chiba University, 1-8-1 Inohana, Chuo-ku, Chiba, Chiba, 260–8670, Japan; 3 Research Center for Child Mental Development, Graduate School of Medicine, Chiba University, 1-8-1 Inohana, Chuo-ku, Chiba, Chiba, 260-8670, Japan

**Keywords:** Anorexia nervosa, Pharmacotherapy, Treatment, Serotonin 1A receptor, Tandospirone

## Abstract

This case report details the therapeutic effects of tandospirone on two patients with anorexia nervosa, one with the restricting subtype (ANR), and another with the binge-eating/purging subtype (ANBP). A 22-year-old female patient with ANR and a 23-year-old female patient with ANBP were treated successfully with the 5-HT1A partial agonist tandospirone. After treatment, not only did both patients gain weight, they also showed improved scores on the Eating Disorder Examination Questionnaire. In addition, a 6-month follow-up showed maintenance of these effects. In this disorder, tandospirone may be an effective drug for long-term use with good patient compliance.

## Introduction

Eating disorders such as anorexia nervosa (AN) have a relatively high incidence in women and the highest mortality rate among mental disorders [1]. Patients suffering from AN show characteristic symptoms, typified by an intense fear of gaining weight or becoming fat, body image distortion, and eating concerns. They are distressed by their obsessive psychopathology. Even when their body mass index falls below minimum levels, they still report feeling larger than they actually are and maintain their fear of gaining weight. Although they have a keen interest in eating, they often restrict calorie consumption and/or vomit after binge eating. In addition, despite significant physical symptoms, patients tend to be in self-denial about the seriousness of their disease. Attempts to treat this disease are confounded by patients frequently presenting with physical problems, such as heart failure, increased susceptibility to infection, and osteoporosis.

There are currently no definitively effective treatments for AN, and although various medications including selective serotonin reuptake inhibitors (SSRIs) or antipsychotics have been evaluated in the past, there is little evidence to support the benefit of psychotropic medication for AN treatment [2]. One reason for this lack of evidence is that robust, randomized, placebo-controlled studies are difficult to perform due to high dropout rates [3]. This lack of compliance may be due to denial about their disease as well as a continued fear of gaining weight. Treatments that target the fear of gaining weight and body image distortion seem to be more acceptable to AN patients and therefore more effective than treatments that solely target weight gain. This is a pivotal issue for consideration in the prevention of relapse and aggravation of symptoms [4].

There are several biological hypotheses about AN that highlight a relationship between disease pathology and serotonin [5]. Neuroimaging studies (e.g., positron emission tomography and single photon emission tomography) have identified a role for the 5-HT1A receptor, and it is already known that upregulated expression of this receptor in various areas of the brain is associated with anxiety [6].

This report aims to show the effects of the 5-HT1A partial agonist tandospirone in the treatment of AN. Both cases showed weight gain in a relatively short time period and improvement in the Eating Disorder Examination Questionnaire (EDE-Q) [7], which is a self-report questionnaire useful for determining the effect of treatment and providing a comprehensive assessment of the behavioral and psychological parameters characteristic of the disorder. The EDE-Q generates four subscales (restraint, eating concern, weight concern, and shape concern) as well as a global score. Higher scores indicate a greater severity of eating disorder psychopathology.

## Case presentation

### Case A

Patient A is a 23-year-old married female with one child. After her husband had an affair with a thinner woman, she decided to lose weight. Subsequent to this, she began to experience nausea and palpitations on eating. Before these symptoms developed, she weighed approximately 50 kg, with a body mass index (BMI) of 20.6. Within 5 months, she weighed 41 kg and was amenorrheic. Her eating patterns were severely restricted to one meal between morning and noon per day.

She presented at our clinic complaining of noncausal nausea and weight loss. At her first visit, she measured 158-cm tall and weighed 41.3 kg, with a BMI of 16.5. At this point, she had already lost 8 kg and had not had menses for almost 4 months. Although she admitted that she had lost weight, she felt that she still looked the same and expressed a desire to reduce her weight further to 30 kg. She also complained about a fear of gaining weight. She was diagnosed in accordance with DSM-IV-TR as having AN of restricted type (ANR).

She was prescribed 1 mg of ethyl lofrazepate to combat her anxiety attacks and palpitations. While this symptom showed small improvement, she maintained her fear of weight gain and eating patterns and showed further weight loss from 41.3 to 40 kg (BMI 16.4). At a weight of 40.0 kg, her EDE-Q subscales of restricting/eating concern/weight concern/shape concern and global scores were 4.6/1.6/3.6/4.88 and 3.67, respectively. She was started on tandospirone because of its fewer side effects on the digestive system relative to SSRIs and its antianxiety effects. Patient A was scheduled for two weekly visits to our clinic to monitor side effects, although no specific psychological treatment was provided. At these visits and in the absence of side effects, tandospirone was initiated at 20 mg and escalated by 20 mg every 2 weeks until it reached 60 mg. It was recommended that she take tandospirone twice daily (when prescribed 20 and 40 mg) or three times daily (when prescribed 60 mg). From the onset of treatment, our patient’s dietary intake gradually increased, and she began to gain weight. In addition, she no longer complained about feelings of fear and guilt. Four weeks into therapy, her weight had increased to 43 kg (BMI 17.7), and her EDE-Q scores reduced, with the subscale scores of restricting/eating concern/weight concern/shape concern and global score being 3.4/0.6/1.2/4.0 and 2.3, respectively. After approximately 20 weeks, her menses resumed. At the 6-month point, she was still adhering to her treatment regime, her EDE-Q diagnostic score was 6, and the subscales restricting/eating concern/weight concern/shape concern and global score were 1.2/0/2.0/3.0 and 1.55, respectively. Also, she had gained weight to reach 45.0 kg with a BMI of 18.5.

### Case B

Patient B is a 22-year-old female. She began to restrict her eating after gaining 20 kg during pregnancy. Soon after this, she drank only water during the day and overate and purged in the evening. Within 6 months, she became amenorrheic.

She presented at our hospital seeking help to curb her binge eating and purging. She was in a pattern of binge eating and purging twice in a day. She felt depressed and irritated that she was not in a good physical condition and unable to be a good parent. At the first visit, she measured 153-cm tall, weighed 35.0 kg, and had a BMI of 15.0. Although she had already experienced a 13-kg weight loss and almost 10 months of amenorrhea, she displayed body image distortion saying ‘If I lose more weight, I will be fine.’ Additionally she stated ‘I feel more confident when losing weight.’ It was clear that her self-esteem was tightly linked to her weight. She was diagnosed according to DSM-IV-TR with AN of binge/purging type (ANBP).

First, she was prescribed 1 mg of ethyl lofrazepate to improve her irritability. This proved effective for her insomnia, but her purging habits gradually deteriorated, and she began cathartic abuse. As a result, her weight fell to 34.8 kg (BMI 14.9). At this weight, the EDE-Q subscales of restricting/eating concern/weight concern/shape concern and global score were 2.4/4.4/4.2/5.25 and 4.06, respectively. Tandospirone was started at 20 mg and escalated by 20 mg at 2-week intervals until it reached 60 mg, as in case A. Four weeks after the initiation of tandospirone, she felt an improvement in her depressive mood, and her weight increased to 36.3 kg (BMI 15.4). At this time, the EDE-Q subscales of restricting/eating concern/weight concern/shape concern and global score were 2.4/2.2/0.2/1.75 and 1.64, respectively. Although her binge eating and purging continued, she stopped cathartic abuse 3 months into treatment and reduced her binge eating and purging to once a day. Her weight increased further to 39.0 kg (BMI 16.7) by week 24, and her EDE-Q subscales of restricting/eating concern/weight concern/shape concern and global score were 2.4/2.0/0.6/3.5 and 2.13, respectively. Shape concern and weight concern subscale scores increased compared to those obtained in week 4. This is thought to be due to the subject being in a bad mood after a patient commented that she looked fatter.

## Discussion

To the best of our best knowledge, this is the first case report showing a therapeutic effect of tandospirone in treating AN of both the restricting and binge/purging types. In the USA and Europe, an equivalent 5-HT1A receptor partial agonist, buspirone, is available on the market, but its effect in treating AN is unknown.

Several studies using positron emission tomography and single photon emission computed tomography with 5-HT-specific radioligands have consistently shown 5-HT1A transporter alterations in AN patients within the cortical and limbic structures, which may be related to behavioral inhibition and body image distortions [5]. Tandospirone mediates two important effects that are integral to alleviating the symptoms of AN. First, tandospirone induces improvement not only in weight gain but also in EDE-Q scores, suggesting improved psychopathology of the patient towards their disorder (Table 1). Disease psychopathology is a core and treatment-refractive symptom of AN. Patients have a morbid fear of weight gain and body image distortion. Treatments that target this core symptom are more likely to be effective in terms of patient compliance and thus prevention of relapse and aggravation of symptoms, compared with treatments that only target weight gain. In these two cases, only psychotropic medication was administered, and no AN-specific psychotherapy was performed. We found that tandospirone improved EDE-Q scoring in both patients. Significant improvements were seen in shape concern and global scores, when comparing week 0 with week 24, using *t* test (Table 1). After treatment, patient A was able to eat an additional afternoon meal, while patient B stopped her cathartic abuse and reduced her binge and purge behavior to once a day. As mentioned above, PET studies have shown that 5-HT1A receptors are highly expressed in specific areas of the brain in patients with AN. This high expression in the dorsal raphe leads to a deterioration of serotonin neuron activity, resulting in a decrease of extracellular serotonin [8]. Tandospirone is a selective agonist of the 5-HT1A receptor [9], and it is thought to desensitize presynaptic 5-HT1A autoreceptors on dendrites of serotonin neurons in the dorsal raphe and thus normalize the function of serotonin neurons [10]. It is also possible that tandospirone may act directly on the 5-HT1A autoreceptor to improve the psychopathology seen in AN.

**Table 1:** Progress in BMI and the EDE-Q of the two cases since administering tandospirone

	**Time after tandospirone (weeks)**						***p *value**^**a**^
	**Case A**			**Case B**			
	**0**	**4**	**24**	**0**	**4**	**24**	
BMI	16.4	17.7	18.5	14.9	15.4	16.7	^*^p = 0.049
Height	156	156	156	153	153	153	
Weight	40.0	43.0	45.0	34.8	36.3	39.0	
EDE-Q							
Restricting subscale	4.6	3.4	1.2	2.4	2.4	2.4	*p* = 0.500
Eating concern subscale	1.6	0.6	0.0	4.4	2.2	2.0	*p* = 0.126
Weight concern subscale	3.6	1.2	2.0	4.2	0.2	0.6	*p* = 0.234
Shape concern subscale	4.88	4.0	3.0	5.25	1.75	3.5	^*^p = 0.023
Global score	3.67	2.3	1.55	4.06	1.64	2.13	^*^p = 0.028

^a^Paired Student’s *t* test, two-tailed alpha level of 0.05 at 0 week versus 24 week. ^*^p < 0.05.

Secondly, tandospirone treatment has a low incidence of side effects. Patients with AN often also suffer from a range of physical problems, such as loss of bone mass or severe obstipation, as a consequence of being in a persistent state of subnutrition. Therefore, the low incidence of side effects, particularly digestive symptoms that interfere with eating or hypnesthesia that may increase the risk of falls, does not add to the patient’s burden. Pharmacologically, tandospirone has a much lower frequency of side effects than the majorly prescribed SSRIs or antipsychotics used in Japan [11-15]. Antipsychotic medication has been proven useful in treating AN [16], but the duration of these studies is relatively short, and the efficacy for relapse prevention in the long term is not proven. In addition, the associated weight gain induced by these drugs leads to high levels of patient noncompliance. To date, there is no evidence that tandospirone induces weight gain. These combined factors make tandospirone an attractive drug for long-term use, with high compliance in this cohort of patients.

This study is by no means conclusive. The relapse rate of AN is very high, and in this study, our patients were followed up for 6 months. This time frame may need to be extended in order to evaluate the effect of recurrence prevention with this drug. Further, controlled trials in AN are needed to fully explore the effects of tandospirone in clinical practice.

## Conclusion

We report cases of patients with ANR and ANBP who were successfully treated with 5-HT1A partial agonist tandospirone. Tandospirone may be an effective drug for long-term use with good patient compliance due to its pharmacological characteristics.

## Consent

Written informed consents were obtained from the patients for publication of this case series and accompanying images. A copy of the written consent is available for review by the Editor-in-Chief of this journal.

## Abbreviations

AN: anorexia nervosa; ANR: anorexia nervosa restricting subtype; ANBP: anorexia nervosa binge-eating/purging subtype

## Competing interests

The authors declare that they have no competing interests.
